# Genetics in Congenital Adrenal Hyperplasia Due to 21-Hydroxylase Deficiency and Clinical Implications

**DOI:** 10.1210/jendso/bvaf018

**Published:** 2025-01-28

**Authors:** Paola Concolino, Henrik Falhammar

**Affiliations:** Dipartimento di Scienze di Laboratorio ed Ematologiche, UOC Chimica, Biochimica e Biologia Molecolare Clinica. Fondazione Policlinico Universitario Agostino Gemelli IRCCS, Roma 00168, Italy; Department of Molecular Medicine and Surgery, Karolinska Institutet, Stockholm SE-171 76, Sweden; Department of Endocrinology, Karolinska University Hospital, Stockholm SE-171 76, Sweden

**Keywords:** congenital adrenal hyperplasia (CAH), 21-hydroxylase deficiency (21OHD), *CYP21A2*, molecular diagnosis

## Abstract

Of all congenital adrenal hyperplasia (CAH), 95% to 99% is 21-hydroxylase deficiency (21OHD), an autosomal recessive disease. 21OHD is due to an insufficiency of 21-hydroxylase enzyme, which is encoded by the *CYP21A2* gene and involved in cortisol and aldosterone production. The clinical presentation differs widely from severe classic to mild nonclassic CAH. 21OHD represents one of the most complex and at the same time intriguing topics in human genetics and its molecular diagnosis involves ongoing challenges. To provide a meticulous presentation of the topic, we searched the past and present literature, including original articles and reviews from PubMed, ScienceDirect, Web of Science, Embase, and Scopus, using search terms for genetics of 21OHD, 21OHD variants, molecular diagnosis of 21OHD, and 21OHD genetic testing. We offer a comprehensive review focusing on recent developments, new concepts, and conclusions.

Congenital adrenal hyperplasia (CAH) is a set of monogenic autosomal recessive disorders resulting from pathogenic variants in genes encoding enzymes and/or proteins in the cortisol biosynthesis pathways: 21-hydroxylase (21OH), 11β-hydroxylase (11βOH), 17α-hydroxylase (17OH; also known as 17,20-lyase), 3β-hydroxysteroid dehydrogenase type 2 (3βHSD2), steroidogenic acute regulatory protein (StAR), P450 cholesterol side-chain cleavage enzyme (SCC), and P450 oxidoreductase (POR) (Fig. 1). Through a negative feedback system, the lack of cortisol synthesis causes an increase in adrenocorticotropic hormone (ACTH) that determines hyperplasia of the adrenal cortex due to the continuous overstimulation and hypersecretion of precursors upstream of the enzyme defect.

**Figure 1.**
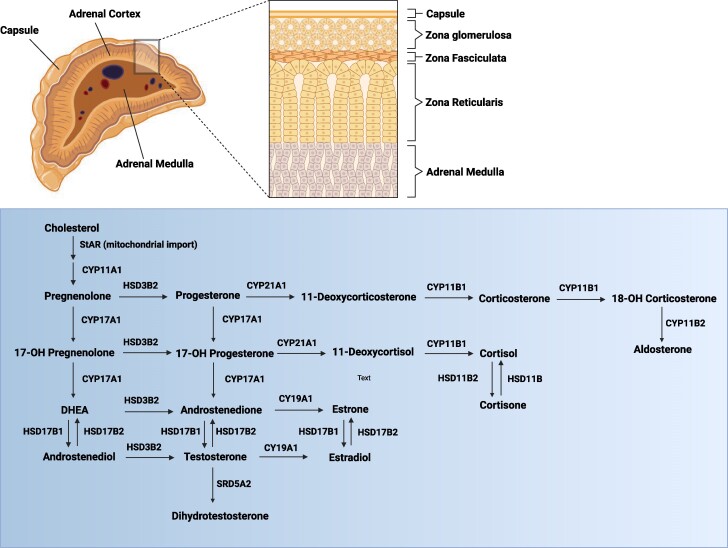
The biochemistry of steroidogenesis. Typically, steroid hormones are classified into 5 groups: glucocorticoids, mineralocorticoids, androgens, estrogens, and progestogens. Adrenal gland is the major source of glucocorticoids (zona fasciculata) and mineralocorticoids (zona reticularis). Cortisol is the major representative of glucocorticoids, while aldosterone is the most prominent mineralocorticoid. The sex hormones—androgens (eg, testosterone and androstenedione), estrogens (eg, estradiol and estrone), and progestogens (such as progesterone)—are mainly synthesized by the gonads and placenta. The bottom of the Figure shows the biosynthetic pathways for major representatives of these classes of steroid hormones. The process of steroidogenesis initiates with the conversion of cholesterol to pregnenolone by cholesterol side-chain cleavage enzyme cytochrome P450scc (CYP11A1) within the mitochondria. Steroidogenic acute regulatory protein (StAR) facilitates the transport of cholesterol within the mitochondria. Pregnenolone is then catalyzed into other steroids by a series of oxidative enzymes located in both mitochondria and endoplasmic reticulum. 17α-hydroxylase (CYP17A1) hydroxylates pregnenolone and converts it to 17α-hydroxypregnenolone. Hydroxy-delta-5-steroid dehydrogenase, 3 beta (HSD3B2) oxidizes pregnenolone and forms progesterone, which is further hydroxylated by 21-hydroxylase (CYP21A2) and forms 11-deoxycorticosterone. All mineralocorticoids are synthesized from deoxycorticosterone. 17α-hydroxyprogesterone is produced from progesterone or from 17α-hydroxypregnenolone. 21-hydroxylase (CYP21A2) converts 17α-hydroxyprogesterone to 11-deoxycortisol from where glucocorticoids (eg, cortisol) are synthesized. 17,20-lyase (CYP17A1) acts on both 17α-hydroxypregnenolone and 17α-hydroxyprogesterone and forms dehydroepiandrosterone and androstenedione, respectively, which act as precursors for testosterone and estrogen biosynthesis. Abbreviations: CYP11B1: cytochrome P450 family 11 subfamily B member 1; CYP11B2, cytochrome P450 family 11 subfamily B member 2; CYP19A1, cytochrome P450 family 19 subfamily A member 1; HSD11B2, hydroxysteroid 11-beta dehydrogenase 2; HSD11B, hydroxysteroid 11-beta dehydrogenase 1; HSD17B1, hydroxysteroid 17-beta dehydrogenase 1; HSD17B2, hydroxysteroid 17-beta dehydrogenase 2; SRD5A2, steroid 5 alpha-reductase 2.

Depending on the type and severity of steroid block, a range of hormonal imbalances occur, and CAH presents with several clinical and biochemical phenotypes, with glucocorticoid production impairment, although to varying degrees [1, 2]. However, the development of adrenal crises depends on the accumulation of steroid precursors with glucocorticoid activity.

The predominant enzyme deficiency that accounts for 95% to 99% of all CAH cases is 21-hydroxylase deficiency (21OHD) [1, 3]. The 21OH enzyme converts 17α-hydroxyprogesterone to 11-deoxycortisol and progesterone to deoxycorticosterone. These steroid precursors are then converted to cortisol and aldosterone (via corticosterone and 18-hydroxycorticosterone). Several studies on adrenal cortex function have established that the zona glomerulosa is responsible for the production of aldosterone, while the zona fasciculata is responsible for the production of cortisol [4]. In 21OHD, all steroid precursors above the block are shunted to adrenal androgens and since the cortisol is low the ACTH secretion from the pituitary gland will increase further shunting steroid precursors to the androgen pathway and the adrenals will be hyperplastic (Fig. 1).

21OHD comprises 2 groups, the classic and nonclassic (NC) form. Classic 21OHD is further divided into salt-wasting (SW) (cortisol and aldosterone deficiency) and simple virilizing (SV) phenotype (cortisol and milder aldosterone deficiency) [5]. The first studies on 21OH activity indicated that the zona fasciculata of both the SW and the SV forms is defective in 21-hydroxylation of 17-hydroxy- and 17-deoxysteroids. The zona glomerulosa demonstrated deficient 21-hydroxylation only in the SW form, whereas in the SV form, the glomerulosa was spared this defect [6-8].

46,XX children with classic CAH have external virilized genitals while both 46,XX and 46,XY children with SW CAH will succumb to adrenal crisis if not diagnosed during the neonatal period. In contrast, 46,XX children with unnoticed virilization, and 46,XY children with SV CAH, may be undiagnosed until later in childhood or even occasionally in adulthood [3, 9]. To prevent fatal SW crisis and late diagnosis in classic CAH, newborn screening (NBS) for 21OHD has been introduced in almost all high-income countries and also in many other countries [2, 10-13]. The NC form is not the aim of NBS; however, approximately one-third of patients with the NC form are incidentally detected during these investigations [12]. Most patients with NC CAH are diagnosed because of symptoms and signs of androgen excess, in childhood to young adulthood [14]. Since adrenal androgen excess will not affect 46,XY adults, most of 46,XY individuals with NC CAH are never diagnosed and if they are, it is usually due to family screening. Among patients with NC CAH, approximately one-third exhibit a partial cortisol response in the ACTH stimulation test [14]. However, these patients rarely experience adrenal crises.

The main therapies in CAH are glucocorticoids to replace the deficiency and normalize the adrenal androgens and mineralocorticoids, compulsory in SW CAH but often also used in SV CAH [2, 15]. Mineralocorticoids, in the form of fludrocortisone, are particularly important prior to (since they may induce ovulation) and during pregnancy in patients with SW CAH but may also be used in those with SV CAH [16, 17]. Unfortunately, to achieve good control of the adrenal androgens, supraphysiological glucocorticoid therapy is usually required, with increased risk of long-term complications such as cardiometabolic diseases as well as osteoporosis and fractures [18, 19]. Moreover, elevated ACTH concentrations and severe phenotype are associated with adrenal rest tumors, especially testicular adrenal rest tumors, which are common and may affect fertility in male individuals with CAH [20, 21]. New therapies to normalize the glucocorticoid doses are, however, on the horizon [15, 22].

The serum 17α-hydroxyprogesterone (17OHP) concentration is utilized as the principal marker for 21OHD, since CAH due to 21OHD is detected in NBS programs using 17OHP concentration measurements in dried blood spots [2]. Genetic testing for 21OHD enzyme defects is currently available in many countries. Molecular diagnosis is important to confirm NBS results [23, 24], for differential diagnosis and for ensuring proper family counseling with carrier identification and risk planning.

Nevertheless, 21OHD represents one of the most complex—and at the same time intriguing—topics in human genetics and its molecular diagnosis involves ongoing challenges. The aim of this review is to summarize the current knowledge about the genetics of 21OHD, with an emphasis on recent data.

## Genetic Background of 21OHD: Gene Locus Structure and Nature of *CYP21A2* Variants

**Figure 2.**
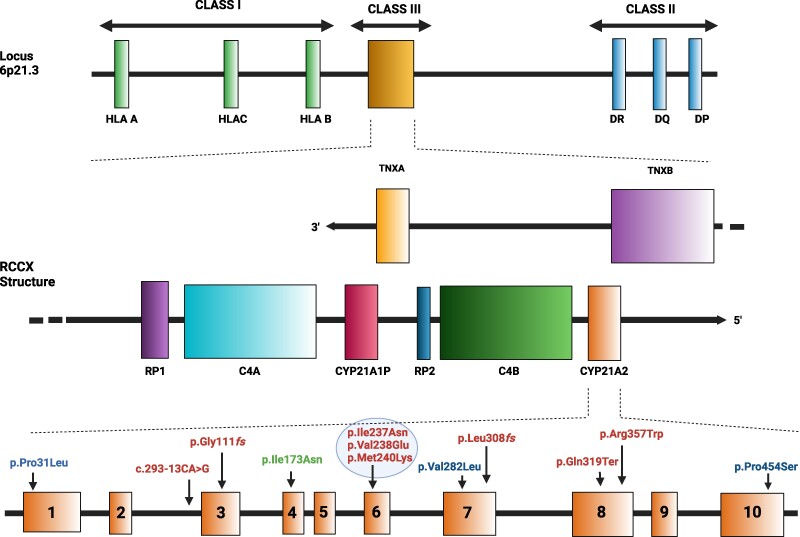
Organization of the human RCCX CNV on chromosome 6 within the HLA class III region. RCCX structure with 2 segments contains the *RP1-C4A-CYP21A1P-TNXA-RP2-C4B-CYP21A2-TNXB* genes, with a telomere-to-centromere orientation. With respect to the *C4* and *CYP21*, both the *TNXA* and *TNXB* genes are in the opposite DNA strand with, consequently, an opposite transcriptional orientation. *CYP21A2* gene consists in a total of 10 exons spanning 3.4 kb. The bottom of the figure shows the most common *CYP21A2* pathogenic variants (red: severe deficit; green: moderate deficit; blue: mild deficit) and their localization in the different exons. Variants are reported in relation to the *CYP21A2* cDNA reference sequence NM_000500.9 and protein reference sequence NP_000491.4.

RCCX copy number variation (CNV), in the class III region of the major histocompatibility complex (MHC), is a complex, multiallelic and tandem CNV defined by the variation in the number of copies of a DNA segment covering 4 adjacent genes: the serine/threonine kinase 19 (*STK19*, also known as *RP1*), the complement 4 (*C4*), the steroid 21-hydroxylase (*CYP21*), and the tenascin-X (*TNX*) genes [25-27]. In the Caucasian population, the RCCX structure with 2 segments occurs in 69%, whereas assemblies with 1 and 2 segments occur 17% and 14%, respectively [28]. Figure 2 illustrates a common RCCX structure containing 2 segments with the following gene order: *RP1-C4A-CYP21A1P-TNXA-RP2-C4B-CYP21A2-TNXB* [29]. The inactive serine/threonine-protein kinase STK19 is encoded by the *STK19* gene (also referred to as *RP1*) (OMIM*604977), just upstream of *C4A*, whereas the *STK19B* pseudogene (alias *RP2*), immediately upstream from the *C4B* gene, consists of only a few bases from the 3′ end of the original *STK19* gene [30]. The 2 isoforms of the fourth serum complement are encoded by the *C4A* (OMIM*120810) and *C4B* (OMIM*120820) genes [28], while the *TNXB* gene (OMIM*600985) produces tenascin-XB, a glycoprotein of the extracellular matrix mainly found in the outer reticular lamina of the basement membrane [31]. The *TNXA* pseudogene contains a 120-bp deletion in exon 35 that produces a premature stop codon and renders the gene nonfunctional [32]. The *TNXA* and *TNXB* genes lie on the opposite DNA strand from *C4* and *CYP21* and therefore have opposite transcriptional orientation (Fig. 2). *CYP21A2* (OMIM*613815), encoding the steroid 21OH enzyme, and *CYP21A1P* pseudogene involve 10 exons spanning 3.4 kb. These genes share 98% sequence homology in exons and around 96% sequence homology in introns [33, 34]. *CYP21A1P* pseudogene is inactive due to the existence of deleterious variants that include 7 missense variants scattered across the 10 exons (NP_000491.4:p.Pro31Leu, p.Ile173Asn, p.Ile237Asn, p.Val238Glu, p.Met240Lys, p.Val282Leu, p.Arg357Trp), an 8-bp deletion within the exon 3 (NP_000491.4:p.Gly111*fs*), a splicing variant in intron 2 (NM_000500.9:c.293-13A/C>G), a frameshift variant in exon 7 (NP_000491.4:p.Leu308*fs*) and a nonsense variant in exon 8 (NP_000491.4:p.Glu319Ter). The cluster of 3 missense variants (NP_000491.4: p.Ile237Asn, p.Val238Glu, p.Met240Lys) in exon 6 is known in the published literature as the “E6 cluster.” In addition, 4 single nucleotide variants (SNVs) in the pseudogene promoter region (NM_000500.9:c.-126C>T, −113G>A, −110T>C, and −103A>G), reduce transcriptional activity to approximately 20% [35].

Nonallelic homologous recombination is the key to the genetic diversity of the RCCX region where the unusual co-presence of genes and pseudogenes with high sequence homology is the cause of frequent misalignments during meiosis that result in large structural rearrangements and in copy number changes. Furthermore, the transfer of relatively short sequences between genes (gene conversion) represents a further element increasing the genetic diversity of the RCCX. The results of these events may be responsible for certain human diseases, for example, 21OHD [36, 37].

## Disease-Causing Variants

Only 5% to 10% of small pathogenic variants affecting the *CYP21A2* gene are not the result of genetic conversions; some of these variants have a founder gene effect and provide important information on population migration [38-41]. Over 95% of deleterious variants leading to 21OHD are attributable to intergenic recombination events where microconversions, consisting in the transfer to *CYP21A2* of deleterious variants usually present in the *CYP21A1P* pseudogene, represent 70% to 75% [42]. A consequence of this phenomenon is that a small group of pathogenic variants with recognized phenotypic effects is detected in all populations (Fig. 2).

**Figure 3.**
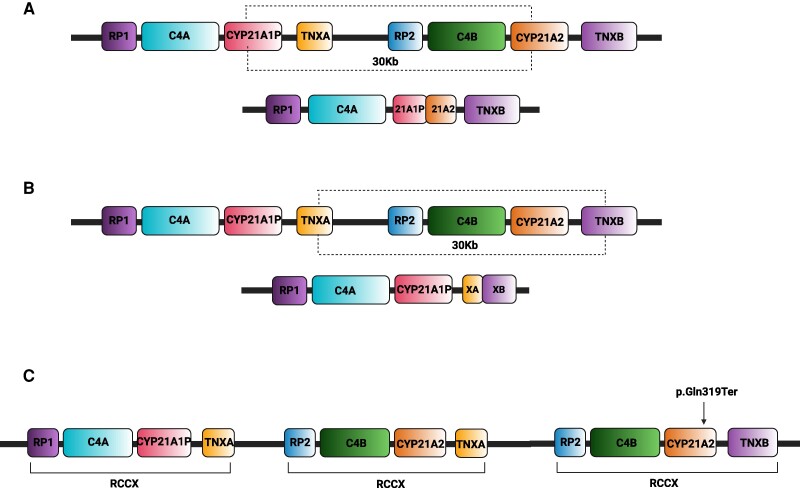
Intergenic recombinations causing genetic rearrangements. (A) *CYP21A1P/CYP21A2* chimeric gene is caused by recombination between *CYP21A1P* and *CYP21A2*. A 30Kb deletion on chromosome 6 causes the impairment of *CYP21A2* and the deletion of *C4B* but leaves safe the *TNXB* gene. Depending on the location of the breakpoint, some chimeras are responsible for the severe salt-wasting form of CAH while others are generally related to a milder phenotype. (B) *TNXA/TNXB* chimeric gene is caused by recombination between *TNXA* and *TNXB*. A 30Kb deletion on chromosome 6 causes the loss of *CYP21A2* gene, the impairment of *TNXB* gene and the deletion of *C4B*. This contiguous deletion is termed CAH-X and causes CAH-X syndrome. (C) RCCX haplotype with 3 segments (*RP1-C4A-CYP21A1P-TNXA-RP2-C4B-CYP21A2-TNXA-RP2-C4B-CYP21A2-TNXB*) carrying a *CYP21A1P* pseudogene and 2 copies of *CYP21A2* gene. Usually, the NP_000491.4:p.Gln319Ter pathogenic variant characterizes the *CYP21A2* copy next to *TNXB* gene.

A nonuniform meiotic crossing-over event is responsible for 20% to 25% of the remaining intergenic recombinations. The most obvious consequences are the deletion or duplication of single genes or larger deletions comprising the *CYP21A2* gene and other adjoining genes [42, 43]. In particular, a single nonfunctional chimeric gene (*CYP21A1P/CYP21A2*), containing the *CYP21A1P* sequence at the 5′ end and the *CYP21A2* sequence at the 3′ end, is the result of an asymmetric recombination event between *CYP21A2* and *CYP21A2P*. In this case, a 30-kb deletion within RCCX region, encompassing the 3′ end of *CYP21A1P*, all the *C4B* gene, and the 5′ end of the *CYP21A2* gene, leaves the *TNXB* gene intact (Figure 3A). Over the years, 2 groups of chimeras, classic and attenuated, have been described: chimeric genes where the junction site is located downstream of the NM_000500.9:c.293-13C/A>G pathogenic variant in the intron 2 belong to the first group. Differently, chimeras carrying the weaker *CYP21A1P* promoter and the sole NP_000491.4:p.Pro30Leu variant, are usually linked to a less severe NC phenotype, although SV phenotypes have also been reported [44-46].

An unequal crossing-over between the *TNXA* and *TNXB* genes can result in the complete deletion of the *CYP21A2* gene. This event, involving a different 30-kb deletion, produces a chimeric *TNXA/TNXB* gene (CAH-X chimera) with the impairment of both *CYP21A2* and *TNXB* genes (Fig. 3B). Different junction sites distinguish several kinds of *TNXA/TNXB* chimeras, determining a condition associated to the CAH-X syndrome [47, 48].

Finally, unequal meiotic crossover is also the cause of *CYP21A2* duplication. In this case, RCCX CNV harbors 3 distinct segments containing 2 *CYP21A2* genes and 1 *CYP21A1P* pseudogene [49] (Fig. 3C). Usually, in this common haplotype, the *CYP21A2* copy next to the *TNXB* gene carries the variant NP_000491.4:p.(Gln319Ter) within exon 8 [50].

## Genotype-Phenotype Correlation

21OHD is inherited in an autosomal recessive manner. The milder of the 2 affected alleles will typically determine the phenotype. Consequently, classical 21OHD phenotype derives from the presence of 2 severely affected alleles, while NC 21OHD derives from the presence of either 2 mild alleles or 1 severe and 1 mild allele (compound heterozygous) [51].

The classification formulated by Speiser et al [52] allows establishment of a genotype-phenotype correlation by distributing the causative variants into 4 different groups based on the residual enzymatic activity established by means of in vitro experiments.

Group 0 or Null includes variants with a complete impairment of enzymatic activity (Deletion/Conversion, NP_000491.4:p.Gly111*fs*, E6 cluster, p.Leu308*fs*, p.Gln319Ter, p.Arg357Trp). Patients who are homozygous or compound heterozygous for these variants present a SW phenotype. Group A includes variants with 0% to 1% residual enzyme activity (NM_000500.9:c.293-13C > G). Patients homozygous for the NM_000500.9:c.293-13C>G variant or compound heterozygous for the NM_000500.9:c.293-13C>G variant and a Null variant show a severe phenotype. Group B contains variants with ∼2% residual enzyme activity (NP_000491.4:p.Ile173Asn). Usually, homozygous patients for the NP_000491.4:p.Ile173Asn variant or compound heterozygous with Groups Null or A variants present with the SV form of 21OHD. Finally, Group C includes variants causing a partial impairment of enzyme activity (∼20%-60% residual activity) (NP_000491:p.Pro31Leu and p.Val282leu). Patients homozygous for these variants or compound heterozygotes with Group 0, A or B variants, frequently present with the NC form of the disorder [52].

Though many studies have demonstrated a strong correlation between genotype and phenotype, multiple reports have found discordance [43, 53-55]. In this regard, it is important to remember that some of the most common variants of the *CYP21A2* gene can cause variable clinical phenotypes. For example, the *CYP21A2* NM_000500.9:c.293-13A/C>G deleterious variant is usually related to SW 21OHD, however, some homozygous or compound heterozygous patients show the less severe SV or even the NC clinical phenotypes. A possible explanation could derive from the fact that in this case the production of a small amount of functional 21OH enzyme is guaranteed by a small number of correctly spliced transcripts. This seems to be sufficient to guarantee a milder clinical presentation of the disease [56].

In contrast, it is not clear why the variants NP_000491.4:p.Ile173Asn and NP_000491.4:p.Pro30Leu, respectively associated with the SV and NC forms, can in some cases give rise to a more severe phenotype: SW (NP_000491.4:p.Ile173Asn) [43, 52, 55] and SV (NP_000491.4:p.Pro30Leu) [57]. For pathogenic missense variants, the lack of genotype-phenotype correlation has been attributed to differences in the 21-hydroxylation of progesterone in the liver by hypermetabolic variants of cytochrome P450 [58]. Another point that could explain differences in the genotype-phenotype correlation in 21OHD is the presence of polymorphic variants in the *POR* gene, the electron donor for 21OH, which reduce its electron transfer capacity [59].

The widespread use of CNV assessment techniques has now clarified some cases of genotype-phenotype discordance found worldwide [60]. In particular, it is now known that a rare RCCX haplotype with 2 copies of the *CYP21A2* gene, one of which carries the pathogenic NP_000491.4:p.Gln318Ter variant, exists in several populations [50].

In this regard, clinical observations confirmed that all subjects with the NP_000491.4:p.Gln318Ter variant on the duplicated gene and a second *CYP21A2* pathogenic variant in *trans* are clinically unaffected [61].

## CAH-X Syndrome

The genetic cause of CAH-X syndrome is a recombination event between *TNXB* and its pseudogene *TNXA*. The resulting 30 kb deletion includes the entire *CYP21A2* gene sequence and part of the *TNXB* gene and produces a chimeric *TNXA/TNXB* gene (CAH-X chimera) (Fig. 3B) [47]. The functional consequence of this rearrangement is the complete absence of the 21OH enzyme and the deficiency of tenascin-X [48, 62], while the resulting phenotype is a hypermobility form of Ehlers-Danlos syndrome (EDS).

Based on the location of the junction site, 3 different *TNXA/TNXB* chimeras have been reported [63]. The *CH1* chimera causes tenascin-X deficiency through a haploinsufficiency mechanism that determines a reduction in protein expression both in the dermis and in the serum [64, 65]. In this case the gene defect consists of a 120-bp deletion, deriving from the pseudogene *TNXA*, within exon 35.

However, CH-2 and CH-3 chimeras carry a single missense *TNXB* variant in exon 40 (NP_061978.6:p.Cys4058Trp) and a cluster of 3 missense variants (NP_061978.6:p.Arg4073His, p.Asp4172Asn, p.Ser4175Asn) in exon 41 and 43, respectively. These 2 chimeras are linked with a dominant-negative effect [64, 66].

The main clinical signs of CAH-X syndrome are linked to connective tissue disorders, namely, musculoskeletal (generalized joint hypermobility, subluxations, chronic arthralgias); dermatological (skin hyperextensibility, abnormal scarring); cardiac (congenital defects, atrioventricular dilation); and/or digestive disorders (hernias, prolapses) [47, 67]. Clinical manifestations of the disorder seem to be more severe in patients with 21OHD; however, it is well documented that subjects carrying *TNXA/TNXB* chimeras also present symptoms of EDS [67]. The prevalence of CAH-X is approximately 11% to 15% in large cohorts of patients with 21OHD, and clinical manifestations of the disease have been described for different populations [48, 64, 68, 69]. Currently, there is no specific therapy for CAH-X syndrome and the clinical diagnosis is performed by detecting related EDS symptoms in patients with 21OHD. Furthermore, to date, CAH-X testing is not standard practice in molecular diagnostic laboratories as it is not regulated by guidelines [70, 71].

## Molecular Diagnosis of 21OHD

Due to the structural complexity of the RCCX region and the frequent rearrangements that occur, genetic tests investigating *CYP21A2* defects must necessarily be guided by specific protocols [71, 72]. To date, polymerase chain reaction (PCR)-based sequence analysis together with the multiplex ligation-dependent probe amplification (MLPA) test represents the best-practice genotyping able to respond to the diagnostic challenge. Because of the high sequence homology between gene and pseudogene, the choice of a winning strategy for the specific amplification of the *CYP21A2* gene appears fundamental.

Two well-defined protocols enable avoidance of errors by ensuring the selective amplification of the *CYP21A2* allele downstream of the *TNXB* gene and of the *CYP21A1P* pseudogene downstream of the *TNXA* gene. In the first case, an 8.5 Kb fragment containing the *CYP21A2* gene is generated, while the amplification of a 6.1 Kb segment permits to isolate the pseudogene [73, 74]. However, it is always necessary to consider that, because of deletions and duplications, the structure of the RCCX region could modify and amplified fragments may contain chimeric genes or duplicated genes (*CYP21A2*-like gene) [72]. MLPA is recommended as the first step in 21OHD molecular diagnosis to determine the exact copy number of genes and pseudogenes within the RCCX region and to make it easier to understand results from the subsequent amplification-sequencing step. In this regard, we would like to point out that the interpretation of MLPA results may prove to be a very difficult challenge in some cases [72].

Approximately 6% to 7% of alleles carry 2 or more pathogenic variants in *cis* [35]. For an accurate genetic diagnosis, it is necessary to perform segregation analysis of these variants in the parents’ DNA samples. In fact, the lack of this analysis could contribute to discrepancies in the molecular diagnosis.

Finally, regarding the application of massive sequencing technologies to the molecular diagnosis of 21OHD, it should be considered that to date, next-generation sequencing (NGS) is not yet recommended for *CYP21A2* genotyping. In fact, NGS, in the current state of the art, can identify pathogenic variants of the pseudogene and contribute to an inadequate molecular diagnosis [75]. However, soon, some third-generation platforms, utilizing direct sequencing of long DNA strands without previous amplification, could represent promising tools [76, 77].

## Prenatal Diagnosis

Pharmacological treatment with dexamethasone of a fetus at risk of CAH must necessarily be initiated prior to the ninth week of gestation. Consequently, the timing of prenatal diagnosis becomes crucial to prevent genital virilization [78]. It should be noted that treatment with dexamethasone to the mother to prevent virilization of the unborn child is controversial and should only be done in an ethically approved follow-up study [2, 3]. Moreover, if no prenatal diagnosis of the fetus is done, only 1 out of 8 fetuses will have the advantage of dexamethasone treatment, while the others only face potential negative effects. Chorionic villus sampling (CVS), performed between 9 and 11 weeks of gestation, with molecular genotyping is currently the favored diagnostic method for prenatal diagnosis of CAH. Alternatively, amniocentesis can be performed in the second trimester, but this procedure has the disadvantage of requiring drug therapy of unaffected fetuses for an extended duration compared to CVS.

In both cases, fetal samples can be amplified using PCR and subsequently sequenced allowing the detection of pathogenic variants and rearrangements causing 21OHD [78]. However, although CVS and amniocentesis are diagnostic of this condition, they are associated with the risk of infections, bleeding, and even miscarriage [79]. In addition, in a minor fraction of the patients undergoing prenatal genetic diagnosis pitfalls do occur, for example, undetectable pathogenic variants, allele dropouts, or maternal DNA contamination [80, 81].

Noninvasive prenatal diagnosis of CAH, which avoids risks related to amniocentesis and CVS, can be performed with fetal cell-free DNA (cff-DNA) that can be traced in the maternal circulation in the first weeks of gestation (fourth or fifth) before the completion of organogenesis [82, 83]. This technique is advantageous in male fetuses as the sex-determining region Y (SRY) sequence can be identified to determine sex as early as the fifth gestational week by using target PCR [84, 85]. Once determination of the fetal sex is done, prenatal treatment should be considered to prevent virilization in affected female fetuses, while if fetal sex is determined to be 46,XY, prenatal treatment is not indicated as complications of virilization would not apply [84, 86]. New et al have developed a succeeding strategy for the noninvasive prenatal diagnosis of 21OHD using cff-DNA extracted from 3.6 mL of maternal plasma. Hybridization probes aimed at capturing a 6-Mb region flanking *CYP21A2* were designed. Targeted massively parallel sequencing (MPS) was utilized to analyze genomic DNA samples from parents and proband and to identify parental haplotypes. In all 14 families, the fetal CAH status was correctly determined by 5 weeks and 6 days of gestation [87].

## Correlation Between Genotype and Long-Term Outcomes

There is some evidence that knowing the genotype of the patient with CAH can predict long-term outcomes. For example, there is a correlation between the severity of the *CYP21A2* genotype and non-heterosexuality in women with CAH as 50% of women with the Null genotype have been reported to have a non-heterosexual orientation. Similar correlations in adult women with CAH were seen between genotype and working in male-dominant occupation and between genotype and motor interest [88]. However, it should be noted that long-term postnatal serum androgen concentrations have not been evaluated in these type of studies [89]. Furthermore, it was observed that the more severe genotype and phenotype in women with CAH the less were the chances of biological children [16, 90]. Such a correlation between genotype severity and having biological children was not seen in males with CAH [91]. It is possible to discuss whether trauma related to atypical genitalia influences lower desire among women with CAH to establish stable relationships and/or to become mothers. However, it should be noted that among women with CAH who wish to become mothers, there were no significant differences between the SW and SV forms [92]. Finally, the correlation between genotype and other long-term outcomes, such as mortality [93], psychiatric diseases [94, 95], cardiometabolic complications [96, 97], autoimmune diseases [98], fractures [19], injuries and accidents [99], were more unclear.

## Concluding Remarks and Future Directions

The genetics of CAH due to 21OHD are complex. However, a genetic test to determine the *CYP21A2* variants is central in confirming the 21OHD diagnosis, genetic counseling, to predict the phenotype and possibly other aspects of CAH such as long-term outcomes. The discovery of CAH-X demands more of the *CYP21A2* gene analysis but will benefit the patients—although more research is needed. Due to the complexity of the gene analysis, simpler and cheaper methods are necessary, especially in countries where resources are more limited [100]. NGS sequencing could be something for the future. The demand for prenatal diagnosis and preimplantation genetic testing is increasing. This latest approach enables studying the embryo before the transference to the uterus and allows prevention of the need to treat an unaffected embryo. This represents a major step forward for a couple to avoid clinical termination of an affected pregnancy [2, 77, 82]. However, most patients with CAH have never had a *CYP21A2* gene analysis, not even in many high-income countries, and wider use should be a priority.

## Data Availability

Data sharing is not applicable to this article as no datasets were generated or analyzed during the current study.

## Abbreviations

ACTH: adrenocorticotropic hormone
CAH: congenital adrenal hyperplasia
CAH-X: congenital adrenal hyperplasia-X (syndrome)
cff-DNA: cell-free fetal DNA
CNV: copy number variation
CVS: chorionic villus sampling
EDS: Ehlers-Danlos syndrome
MLPA: multiplex ligation-dependent probe amplification assay
NBS: newborn screening
NC: nonclassic
NGS: next-generation sequencing
PCR: polymerase chain reaction
SV: simple virilizing
SW: salt-wasting
21OH: 21-hydroxylase
21OHD: 21-hydroxylase deficiency
